# Eating disorders are psychiatric illnesses heavily burdened by death risk

**DOI:** 10.1192/j.eurpsy.2026.10349

**Published:** 2026-07-31

**Authors:** P. Courtet

**Affiliations:** University of Montpellier, Montpellier, France

## Abstract

**Abstract:**

Eating disorders (EDs) are among the most lethal psychiatric illnesses, characterized by a markedly elevated standardized mortality ratio compared to the general population. Mortality arises from both severe medical complications—particularly in anorexia nervosa—and suicide, which accounts for a substantial proportion of deaths across diagnostic subtypes. Despite this well-documented risk, suicide vulnerability in EDs remains insufficiently integrated into routine clinical assessment and care pathways.

Recent work by Sébastien Guillaume and colleagues has significantly advanced our understanding of suicidal risk in ED populations. Their findings highlight the central role of affective dysregulation, impulsivity (especially in binge–purge phenotypes), depressive comorbidity, and history of suicide attempts as key drivers of mortality risk. Beyond categorical diagnoses, dimensional constructs such as emotional pain, cognitive rigidity, and stress sensitivity appear crucial in explaining why only a subgroup of patients progresses from ideation to suicidal behavior.

From a precision suicidology perspective, EDs represent a paradigmatic case of dual vulnerability: metabolic fragility interacting with psychiatric and psychological risk trajectories. Not all patients with EDs share the same death risk. Stratification models integrating clinical phenotype (restrictive vs. binge–purge), prior suicidal behavior, depressive severity, and potentially biological markers (e.g., inflammatory or stress-related indicators) may improve identification of high-risk individuals.

Importantly, weight restoration alone does not equate to reduced suicide risk. The post-hospitalization period may constitute a critical window for targeted intervention. Precision-based approaches combining careful medical monitoring, systematic suicide risk assessment, and tailored psychotherapeutic strategies are therefore essential.

Recognizing eating disorders as illnesses with heterogeneous but potentially extreme mortality risk shifts the clinical objective: from symptom management alone to proactive, stratified, and suicide-informed care. Such an approach may ultimately reduce preventable deaths in this vulnerable population.

**Disclosure of Interest:**

None Declared

